# The Fidelity of Artificial Intelligence to Multidisciplinary Tumor Board Recommendations for Patients with Gastric Cancer: A Retrospective Study

**DOI:** 10.1007/s12029-023-00967-8

**Published:** 2023-09-13

**Authors:** Yong-Eun Park, Hyundong Chae

**Affiliations:** 1 Department of Surgery, College of Medicine, Yeungnam University, 170 Hyeonchungno, Nam-gu, Daegu, 42415 Korea; 2 Department of Surgery, School of Medicine, Catholic University of Daegu, 33, Duryugongwon-ro 17-gil, Nam-gu, Daegu, Republic of Korea

**Keywords:** Artificial intelligence, Concordance rate, Gastric cancer, Multidisciplinary team board, Watson for Oncology

## Abstract

**Purpose:**

Due to significant growth in the volume of information produced by cancer research, staying abreast of recent developments has become a challenging task. Artificial intelligence (AI) can learn, reason, and understand the enormous corpus of literature available to the scientific community. However, large-scale studies comparing the recommendations of AI and a multidisciplinary team board (MTB) in gastric cancer treatment have rarely been performed. Therefore, a retrospective real-world study was conducted to assess the level of concordance between AI and MTB treatment recommendations.

**Methods:**

Treatment recommendations of Watson for Oncology (WFO) and an MTB were retrospectively analyzed 322 patients with gastric cancer from January 2015 to December 2018 and the degree of agreement between them was compared. The patients were divided into concordance and non-concordance groups and factors affecting the concordance rate were analyzed.

**Results:**

The concordance rate between the AI and MTB was 86.96%. The concordance rates for each stage were 96.93% for stage I, 88.89% for stages II, 90.91% for stage III, and 45.83% for stage IV, respectively. In the multivariate analysis, age (*p*-value = 0.000), performance status (*p*-value = 0.003 for performance score 1; *p*-value = 0.007 for performance score 2; *p*-value = 0.000 for performance score 3), and stage IV (*p*-value = 0.017) had a significant effect on concordance between the MTB and WFO.

**Conclusion:**

Factors affecting the concordance rate were age, performance status, and stage IV gastric cancer. To increase the validity of future medical AI systems for gastric cancer treatment, their supplementation with local guidelines and the ability to comprehensively understand individual patients is essential.

**Supplementary Information:**

The online version contains supplementary material available at 10.1007/s12029-023-00967-8.

## Introduction

While the amount of medical literature that needs to be covered is increasing rapidly every day, the amount of time available to medical specialists is limited. To account for this limitation, the medical field is subdivided and specialized, and a multidisciplinary team board (MTB) has been introduced for smooth communication between specialists in each subdivided medical field. The MTB is a physical or virtual meeting in which different specialists converge to discuss clinical cases and determine the best diagnostic or therapeutic method. An MTB is recommended in a variety of cancer guidelines and is the principal approach for managing cancer patients in many countries [1].

However, owing to the rapid expansion of medical literature and advances in techniques, staying aware of updates is challenging, even for specialists in subdivided medical fields [2]. Thus, it will become increasingly difficult for MTB specialists to present individualized treatment recommendations for cancer patients based on the latest medical research. In this situation, the application of artificial intelligence (AI) in clinical practice could be an ideal solution for assisting MTB in managing patients with cancer.

AI can support clinicians in the diagnosis, treatment, and prognosis of a variety of diseases through the quick analysis of complex medical data. Therefore, AI has the potential to compensate for human shortcomings and provide better treatment recommendations [3, 4]. Furthermore, reliance on AI in the management of cancer patients is expected to increase over time. However, for AI to be applied in actual medical practice, it must be validated using a pre-existing principal method.

To investigate the validity of AI in managing cancer patients, previous studies have compared the treatment recommendations of an MTB and AI and analyzed the concordance rate. AI has shown a consistent and high concordance rate of 80–90% with an MTB for breast and colorectal cancer in many countries. In contrast, the concordance rate of AI for gastric cancer varies according to country [5]. In addition, unlike other cancers, there are only a few studies on using AI for the treatment of gastric cancer, and these were limited as they only included advanced stages of gastric cancer or the sample size was very small for each stage [6–8].

Therefore, we performed a large-scale study to investigate factors that lower the concordance rate between medical AI and an MTB in the recommendation of gastric cancer treatment. If these factors are supplemented, it is expected that AI will be able to better assist the MTB as one of the members in actual clinical practice.

## Methods

### Patients and Study Design

Treatment recommendations for 322 consecutive gastric cancer cases were analyzed and the degree of agreement was compared between Watson for Oncology (WFO) (IBM Watson Health, Cambridge, MA) and a 7-member MTB comprising two surgeons, two medical oncologists, a pathologist, a radiologic oncologist, and a radiologist at the Daegu Catholic University Medical Center (DCUMC), Daegu, South Korea, from January 2015 to December 2018. Patients who fulfilled the following criteria were eligible for this study: (1) diagnosed with gastric cancer and the results confirmed using gastroduodenoscopy and histologic examination of the resected specimens, and (2) surveillance or adjuvant chemotherapy after curative gastrectomy, or palliative chemotherapy in stage IV. The exclusion criteria were as follows: (1) neoadjuvant chemotherapy or conversion surgery; (2) history of other malignancies within 5 years; (3) incomplete data; and (4) below 18 years of age. The gastric cancer stage was determined according to the 7th edition of the American Joint Commission on Cancer (AJCC). The clinicopathological characteristics of the patients are summarized in Table 1. Patients with stage IV disease progression following systemic therapy (second-line and beyond) were also excluded. The study design was reviewed and approved by the Institutional Review Board (IRB) of DCUMC (DCMC-CR-21-017).

**Table 1:** Baseline characteristics of patients

Variables		(*n* = 322) *n*(%) or mean(SD)
Age (years)	Total	64.43(12.42)
	<80	285(88.5)
	≥80	37(11.5)
Sex	Female	95(29.5)
	Male	227(70.5)
Performance status	0	227(70.5)
	1	44(13.7)
	2	26(8.1)
	3	25(7.8)
Tumor location	Proximal	80(24.8)
	Middle	80(24.8)
	Distal	162(50.3)
HER2 status	Negative	291(90.4)
	Positive	31(9.6)
Lympho-vascular invasion	No	200(69)
	Yes	90(31)
	Not available^a^	32
Perineural invasion	No	200(69)
	Yes	90(31)
	Not available^a^	32
Serum bilirubin (mg/dL)	Total	0.57(0.49)
	<1.2	306(95.0)
	≥1.2	16(5.0)
Serum creatinine (mg/dL)	Total	0.75(0.23)
	<0.9	238(73.9)
	≥0.9	84(26.1)
Concordance	No	42(13.0)
	Yes	280(87.0)
Cancer stage	I	163(50.6)
	II	45(14)
	III	66(20.5)
	IV	48(14.9)

*SD* standard deviation, *HER2* human epidermal growth receptor 2

^a^Not available: data from patients with palliative chemotherapy

### AI and MTB Concordance Determination

The WFO version 16.4 was used as the medical AI. In gastric cancer treatment, WFO considers not only the disease stage, postoperative pathologic findings, human epidermal growth factor receptor 2 (HER2) status, and the patient’s general condition (critical disease and performance status) but also the age at diagnosis, sex, weight, histologic type, and prior therapy.

WFO provides therapeutic recommendations in three categories: recommended, for consideration, and not recommended. Data were retrospectively analyzed to compare the treatment recommendations of the MTB and AI. MTB recommendations were defined as concordant with WFO if they corresponded to the recommended or for consideration categories and as non-concordant if they corresponded to the not recommended or not available categories. Therapies recommended by the MTB were classified as “not available” if they were not known to the WFO at the time of analysis.

### Data Analysis and Statistics

All statistical analyses were performed using SPSS (version 22.0; IBM Corporation, Armonk, NY, USA), with a significance level set at *p* < 0.05. Concordance was expressed as a percentage agreement. Continuous variables are expressed as mean (standard deviation) and compared using a two-sample *t*-test. Categorical variables were expressed as frequencies (percentage) and analyzed using the chi-square test. A logistic regression model was used to simultaneously control for the determinants of concordance, and odds ratios and 95% confidence intervals were calculated.

## Results

**Fig. 1:**
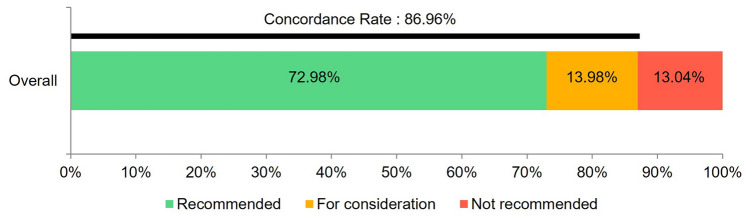
Overall concordance rate between AI and MTB. The concordance rate between WFO and MTB was 72.98% at the recommended level (235/322) and 86.96% at the consideration level (280/322). Abbreviations: AI, artificial intelligence; MTB, multidisciplinary tumor board

The mean age of the 322 gastric cancer patients was 64.43 years (SD, 12.42). Among them, 285 (88.5%) patients were under 80 years of age and 37 (11.5%) were over 80 years old. The distribution of gastric cancer stages was 163 (50.6%) in stage I, 45 (14%) in stage II, 66 (20.5%) in stage III, and 48 (14.9%) in stage IV (Table 1). The concordance rate between WFO and the MTB was 72.98% at the recommended level (235/322) and 86.96% at the consideration level (280/322) (Fig. 1).

In univariate analysis, there were significant differences in age (*p* = 0.03), sex (*p* = 0.016), performance status (*p* = 0.012), tumor location (*p* = 0.014), and gastric cancer stage (*p* < 0.001) between the concordance and non-concordance groups. In contrast, there was no significant difference in HER2 status (*p* = 0.558), lympho-vascular invasion (*p* = 0.224), perineural invasion (*p* = 0.468), serum bilirubin level (*p* = 0.408), and serum creatinine level (*p* = 0.251) between the concordance and non-concordance group (Table 2).

**Table 2:** Univariate analysis of concordance by variables using chi-square test

Variables		Concordance (*n* = 280)	Non-concordance (*n* = 42)	*p*-value
Age	Total	63.71(12.22)	69.21(12.81)	0.007*
	<80	252(90.0%)	33(78.6%)	0.030*
	≥80	28(10.0%)	9(21.4%)	
Sex	Female	76(27.1%)	19(45.2%)	0.016*
	Male	204(72.9%)	23(54.8%)	
Performance status	0	205(73.2%)	22(52.4%)	0.012*
	1	34(12.1%)	10(23.8%)	
	2	23(8.2%)	3(7.1%)	
	3	18(6.4%)	7(16.7%)	
Tumor location	Proximal	71(25.4%)	9(21.4%)	0.014*
	Middle	62(22.1%)	18(42.9%)	
	Distal	147(52.5%)	15(35.7%)	
HER2 status	Negative	252(90.0%)	39(92.9%)	0.558
	Positive	28(10.0%)	3(7.1%)	
Lympho-vascular invasion	No	188(69.9%)	12(57.1%)	0.224
	Yes	81(30.1%)	9(42.9%)	
	Not available^a^	32		
Perineural invasion	No	187(69.5%)	13(61.9%)	0.468
	Yes	82(30.5%)	8(38.1%)	
	Not available^a^	32		
Serum bilirubin (mg/dL)	Total	0.58(0.50)	0.52(0.38)	0.490
	<1.2	265(94.6%)	41(97.6%)	0.408
	≥1.2	15(5.4%)	1(2.4%)	
Serum creatinine (mg/dL)	Total	0.73(0.18)	0.85(0.42)	0.090
	<0.9	210(75.0%)	28(66.7%)	0.251
	≥0.9	70(25.0%)	14(33.3%)	
Cancer stage	I	158(56.4%)	5(11.9%)	0.000*
	II	40(14.3%)	5(11.9%)	
	III	60(21.4%)	6(14.3%)	
	IV	22(7.9%)	26(61.9%)	

^*^Statistically significant at *p* < 0.05

*HER2* human epidermal growth receptor 2

^a^Not available: data from patients with palliative chemotherapy

**Fig. 2:**
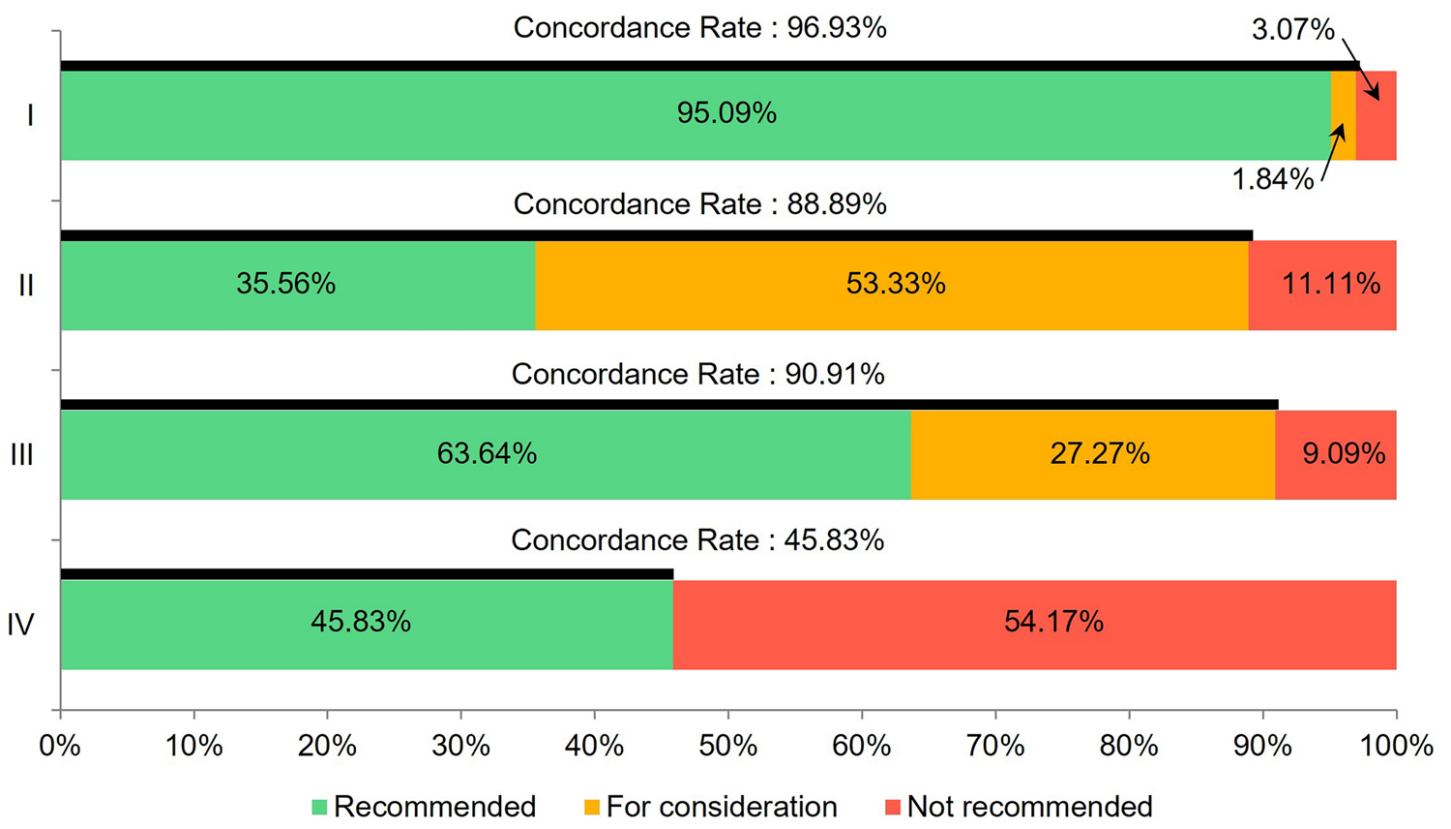
Concordance rate between AI and MTB by gastric cancer stage. The concordance rate for stage I was the highest (96.93%). The concordance rates for stages II and III were 88.89% and 90.91%, respectively, which were close to 90%; however, the concordance rate for stage IV was the lowest at 45.83%. Abbreviations: AI, artificial intelligence; MTB, multidisciplinary tumor board

More than half of the concordance group (56.4%) had stage I disease, and the concordance rate for stage I was the highest at 96.93%. The concordance rates for stages II and III were 88.89% and 90.91%, respectively. However, the concordance rate for stage IV was the lowest at 45.83% (Fig. 2).

**Fig. 3:**
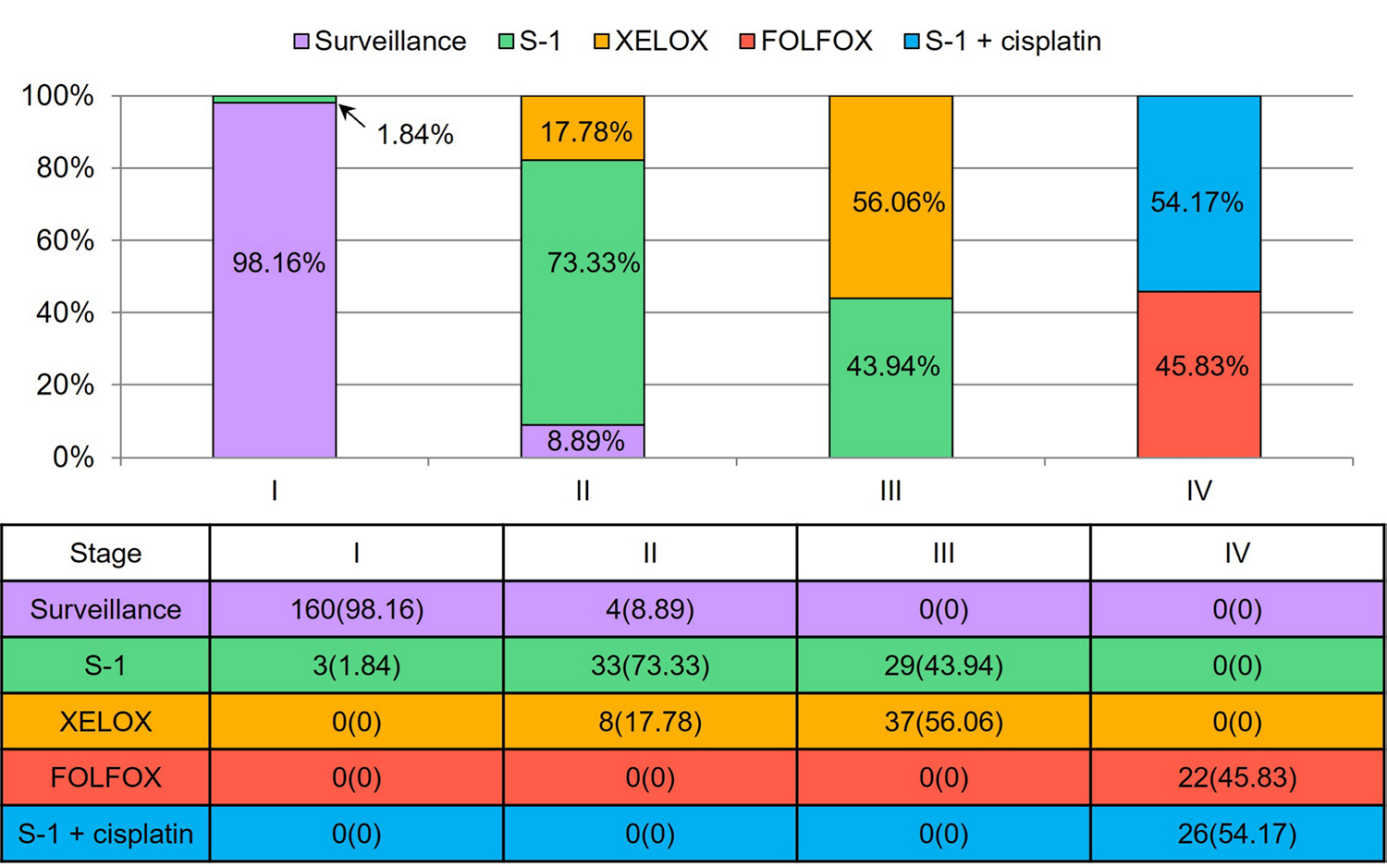
Selected treatment options by gastric cancer stage. All patients with stage I to stage III gastric cancer underwent curative gastrectomy. Of the 48 patients with stage IV gastric cancer, 32 patients were treated with palliative chemotherapy, of which 21 cases were treated with palliative S-1 + cisplatin chemotherapy, and 11 cases with palliative FOLFOX chemotherapy. The remaining 16 patients with stage IV gastric cancer underwent adjuvant chemotherapy after gastrectomy; MTB selected adjuvant FOLFOX chemotherapy for 11 patients and adjuvant S-1 + cisplatin chemotherapy for 5 patients. Abbreviations: MTB, multidisciplinary tumor board; XELOX, capecitabine/oxaliplatin; FOLFOX, fluorouracil/leucovorin/oxaliplatin

The treatment options selected for each stage of gastric cancer were as follows (Fig. 3): All patients with stage I to stage III gastric cancer underwent curative gastrectomy. Among 163 gastric cancer patients with stage I disease, the MTB selected surveillance for 160 patients and S-1 adjuvant chemotherapy for three patients. The selected treatment options for the 45 stage II gastric cancer patients were as follows: surveillance for four patients, S-1 adjuvant chemotherapy for 33 patients, and XELOX adjuvant chemotherapy for eight patients. The selected treatment options for 66 patients with stage III gastric cancer were XELOX adjuvant chemotherapy for 37 patients and S-1 adjuvant chemotherapy for 29 patients. Of the 48 patients with stage IV gastric cancer, 32 patients were treated with palliative chemotherapy, of which 21 cases were treated with palliative S-1 + cisplatin chemotherapy, and 11 cases with palliative FOLFOX chemotherapy. The remaining 16 patients with stage IV gastric cancer underwent adjuvant chemotherapy after gastrectomy, and the MTB selected adjuvant FOLFOX chemotherapy for 11 patients, and adjuvant S-1 + cisplatin chemotherapy for five patients.

The discrepancies between the MTB and AI for each gastric cancer stage were as follows (supplementary tables): In stage I, there were five patients in the non-concordance group for which the AI recommended adjuvant chemotherapy as the treatment option (after gastrectomy) but the MTB selected surveillance (Table S1). In stage II, five gastric cancer patients belonged to the non-concordance group. The AI recommended adjuvant chemotherapy for four patients whereas the MTB selected surveillance as the treatment option. For the remaining gastric cancer patient, AI recommended FOLFOX as adjuvant chemotherapy, but the MTB selected S-1 adjuvant chemotherapy (Table S2). In stage III, there were six patients in the non-concordance group. The AI recommended 5-FU or FOLFOX for four patients, but the MTB selected S-1 adjuvant chemotherapy. For the remaining two patients, AI recommended S-1, capecitabine + radiation, or capecitabine + cisplatin, but the MTB selected the XELOX regimen (Table S3). In stage IV, 26 patients with the S-1 plus cisplatin regimen belonged to the non-concordance group (Table S4).

Table 3 indicates the results of the multivariate analysis of concordance as a function of age, sex, performance status, tumor location, and cancer stage. Several variables, such as age, performance status, and stage IV gastric cancer, had a significant effect on the concordance between the MTB and AI (Table 3).

**Table 3:** Multivariate analysis of concordance using a binary logistic regression model

Variables		*OR*	95% CI for *OR*	*p*-value
Age (ref: <80)	≥80	0.175	0.069, 0.441	0.000*
Sex (ref: female)	Male	2.006	0.916, 4.391	0.082
Performance status (ref: 0)	1	0.203	0.072, 0.574	0.003*
	2	0.191	0.057, 0.639	0.007*
	3	0.089	0.026, 0.301	0.000*
Tumor location (ref: distal)	Middle	1.058	0.377, 2.969	0.914
	Proximal	0.778	0.311, 1.943	0.590
Cancer stage (ref: I)	II	0.367	0.111, 1.220	0.367
	III	0.673	0.218, 2.078	0.673
	IV	0.017	0.005, 0.055	0.017*

^*^Statistically significant at *p* < 0.05

*OR* odds ratio, *CI* confidence interval

## Discussion

This retrospective study was conducted to investigate the factors that should be supplemented in AI to assist the MTB in gastric cancer treatment recommendations. Few studies have analyzed the concordance rate between AI and an MTB in gastric cancer treatment recommendations. Choi et al. reported that stage IV gastric cancer was the only significant factor that affected concordance rates [6]. In contrast, Tian et al. reported that HER-2 positivity was a significant factor, while stage IV was not [8]. Interestingly, in the present study, HER-2 positivity was not significant, but age > 80 years, performance status, and stage IV gastric cancer were all significant factors affecting the concordance rate between the MTB and AI (Table 3).

Recommendations pertaining to patients aged > 80 years were less likely to be concordant than those pertaining to patients aged < 80 years (*OR* 0.175, 95% CI, 0.069–0.441; *p* = 0.000). Elderly patients have more comorbidities than younger patients, a tendency to refuse chemotherapy requiring hospitalization, and a fluctuating general status [9, 10]. These clinical features could explain the lower concordance rate in patients over 80 years of age.

The higher the performance score, the lower the probability of concordance between the MTB and AI in gastric cancer recommendations (*OR* 0.203, 95% CI 0.072–0.574, *p*-value = 0.003 for performance score 1; *OR* 0.191, 95% CI 0.057–0.639, *p*-value = 0.007 for performance score 2; *OR* 0.089, 95% CI 0.026–0.301, *p*-value = 0.000 for performance score 3). In other words, as the performance status score increases, it becomes easier to select a chemotherapy regimen that does not match the AI recommendation. In general, elderly patients have higher performance scores than younger patients [11].

Age > 80 years, performance status were the main factors for discrepancies in stage II and stage III. In stage II, the MTB selected surveillance as the treatment option for four patients, when taking age, performance status score, and comorbidities into account. The patients were aged 68, 69, 72, and 85 and their performance status scores were all grade three. They also had comorbidities, such as dementia, chronic kidney disease, and chronic heart failure. For the remaining gastric cancer patient, the MTB selected S-1 adjuvant chemotherapy because of the inconvenience of frequent hospitalization due to the old age of the patient (82 years) and a performance status score of grade two (Table S2). In stage III, the MTB selected S-1 adjuvant chemotherapy for four patients after considering age (all over 80 years), a performance status score of two or three, and the inconvenience of frequent hospitalization. For the remaining two patients, the MTB selected the XELOX regimen after taking age (49 and 64 years), a performance status of one, and the presence of no comorbidities into account (Table S3).

In this study, there was no significant difference in concordance rates between stage II and stage III when compared to stage I (*p* = 0.367 for stage II, *p* = 0.673 for stage III). Interestingly, the concordance rate for stage IV was significantly lower than that of stage I due to differences in the preference for a palliative chemotherapy regimen in the local guidelines between the MTB and WFO (*OR* 0.017, 95% CI 0.005–0.055, *p* = 0.017). S-1 plus cisplatin is a commonly used chemotherapy regimen in Korea and Japan, following the Japanese guidelines [12, 13], whereas S-1 is an investigational agent in the National Comprehensive Cancer Network (NCCN) guidelines of 2018, and therefore not used in the Memorial Sloan Kettering Cancer Center (MSKCC) following the NCCN guidelines [14]. Therefore, since S-1 + cisplatin was not included in the WFO chemotherapy regimen based on the MSKCC data, the concordance rate between the MTB and AI was significantly lower in stage IV (Table S4). Theses local guideline differences were a primary cause of non-concordance not only in stage IV but also in stage I. D2 lymph node dissection is commonly performed in East Asia, unlike the Western world, and observation is recommended in the Japanese guidelines for pathologic stage I after curative gastrectomy [13]. The MTB judged that since five patients with gastric cancer stage I in the non-concordance group had undergone D2 lymph node dissection and were over 65 years of age, there was little benefit from adjuvant chemotherapy considering its complications (Table S1).

In summary, when analyzing the pattern of discordance between AI and MTB for each stage, along with the results of multivariate analysis, discrepancies due to local guideline differences were primarily observed in stage I and stage IV, while discrepancies based on age > 80 years and performance status were mainly evident in stage II and stage III.

AI is applied in various areas of medicine, such as robotics, medical diagnosis, and medical statistics. WFO, an AI system for clinical decision support, is expected to offer many advantages, such as increased work efficiency and a decreased workload for doctors, decision support for junior oncologists, and treatment selection based on the latest medical research, even in hospitals with few or no experts [15, 16]. However, several factors lowered the concordance rate between the medical AI and experts in gastric cancer, resulting in the reduced validity of the medical AI.

First, AI lacks a comprehensive understanding of individual patient. The WFO cannot understand the comprehensive status of patients, such as patient compliance and rapport with doctors, comorbidities that may affect chemotherapy, and interpretation of whether biochemical study results are temporary or persistent [5].

However, it is expected that these AI shortcomings will be compensated for as technology advances. For example, a wearable device or sensor, that can check the patient’s condition and evaluate their activity 24 h a day, could provide continuous rather than fragmentary patient information. Therefore, an accurate individual performance status can be obtained through individual activity history, rather than through performance scores, which are limited in their range [17, 18]. The development and usage of health applications that can be used on portable computing devices, such as smartphones, are also expanding. If the medical information recorded on a personal device can be easily linked to the medical information database of a country or hospital, the patient’s medical history and comorbidities can be easily identified and applied in clinical practice [19, 20]. In addition, AI assistance for emotional support, such as a robot companion for the elderly with limited cognitive function or activity, is expected [21].

Second, the local guidelines for gastric cancer differ according to race, country, and region. WFO, the medical AI used in this study, is based on the data of MSKCC in the USA. However, gastric cancer treatment in Korea follows the Korean guidelines, which are closer to the Japanese guidelines than the NCCN guidelines [12]. Local guidelines differ in their preferred surgical methods, the effectiveness of specific chemotherapy regimens or radiation therapy, and the approval status of chemotherapy drugs. In the future, besides resolving these local guideline differences, AI may assist in determining the best treatment plan tailored to an individual cancer patient.

Third, there are several economic factors. Owing to the high cost of cancer medicines, an individual patient’s financial circumstances, such as public or private insurance coverage, can affect the chemotherapy regimen choice. Therefore, lowering the price of chemotherapy drugs and expanding insurance coverage will enhance the affordability and accessibility of cancer medicines [22]. AI increases the efficiency of clinical trials and research, thereby significantly lowering the cost of drug development, which in turn lowers the price of cancer medicine [23]. The improved cost efficiency of chemotherapy drugs is also expected to have a positive impact on social discussions and government approval regarding the insurance coverage of anticancer drugs.

In this study, the WFO version 16.4 was used as the medical AI. However, there are various types of AI applicable to the medical field, and ChatGPT is a prominent example of that. ChatGPT, an AI chatbot with remarkable text comprehension capabilities, was released for public use in November 2022. ChatGPT has become a worldwide sensation for its ability to comprehend and respond to questions on a variety of topics. Research on the capabilities and usefulness of ChatGPT has been conducted across various fields, and the medical field is no exception. Brian Schulte conducted a study comparing the ability of ChatGPT to suggest appropriate systemic therapies for 51 different prompts utilizing 32 advanced solid tumors with the NCCN guidelines. The overall ratio of those medications listed by ChatGPT to those suggested in the NCCN guidelines was 0.77 [24]. Georges et al. retrospectively investigated the effectiveness of ChatGPT in assisting healthcare providers with decision-making in the emergency room, focusing on patient with metastatic prostate cancer and concluded that ChatGPT has the potential to assist healthcare providers in enhancing patient triage and improving the efficiency and quality of care in emergency room [25].

However, not only positive results but also significant drawbacks for ChatGPT to function as a medical supporting AI have been reported. ChatGPT can provide varying answers to the same question without providing references, and it can generate incorrect answers in a confident sound manner [26]. Therefore, Zhou et al. reported that we should learn to utilize ChatGPT without relying on it and always remember that it is a chatbot, not a person [27].

Despites these drawbacks, considering the potential of AI shown by ChatGPT and high concordance rate between WFO and MTB, further research and corresponding improvements in capabilities are expected to enable AI to perform well in the role of a medical assistant.

This study had several limitations. First, it was a retrospective and single-center analysis; therefore, it may have been biased. Second, the results of this study were analyzed based on a treatment consensus for WFO and an MTB from 2015 to 2018. If we compare the concordance rates between the last version of WFO and the MTB, based on the latest guidelines, the results may differ. Third, this study only used the concordance rate as a method for evaluating the validity of AI. Therefore, factors such as the extent to which AI influences doctors’ decisions, if better outcomes such as increased overall survival or disease-free survival can be achieved with AI-recommended treatments, and time and cost saving gained by using AI, have not been examined. These factors should be analyzed not only for the validation of AI but also in terms of the usefulness and economic feasibility of AI. However, to investigate these factors, a large-scale prospective study is required, and discussions on the ethics and legal responsibility of AI decisions should be conducted before such a study.

In this study, the factors affecting the dis-concordance between AI and the MTB were age, performance status, and stage IV gastric cancer. The effect of gastric cancer stage IV occurred because of differences in the local guidelines between AI and the MTB. Also, the effects of age and performance status were caused by the inability of AI to comprehensively understand individual patients.

## Conclusion

By taking into account the local cancer guidelines and developing the ability to understand the comprehensive status of cancer patients, a medical AI may be able to not only effectively assist the MTB in the management of gastric cancer patients in actual clinical practice but also play a role as a member of the MTB.

### Supplementary Information

Below is the link to the electronic supplementary material.

Supplementary file1 (DOCX 22 KB)

## Data Availability

The anonymized data used and/or analyzed during the current study are available from the corresponding author upon reasonable request.

## Abbreviations

AI: Artificial intelligence
MTB: Multidisciplinary team board
WFO: Watson for Oncology
DCMC: Daegu Catholic University Medical Center
HER2: Human epidermal growth receptor 2
XELOX: Capecitabine/oxaliplatin
FOLFOX: Fluorouracil/leucovorin/oxaliplatin
OR: Odds ratio
CI: Confidence interval
NCCN: National Comprehensive Cancer Network
MSKCC: Memorial Sloan Kettering Cancer Center
