# Supplementary material for: The Fidelity of Artificial Intelligence to Multidisciplinary Tumor Board Recommendations for Patients with Gastric Cancer: A Retrospective Study
Source: J Gastrointest Cancer. 2023 Sep 13;55(1):365–72. doi: 10.1007/s12029-023-00967-8 (PMC11096204; doi:10.1007/s12029-023-00967-8)
Supplement: Supplementary file 1 — Supplementary file1 (DOCX 22 KB) [file 12029_2023_967_MOESM1_ESM.docx]

**Supplementary Table legends**

**Supplementary table 1.** The discrepancies between the MTB and AI in gastric cancer stage Ⅰ

**Supplementary table 2.** The discrepancies between the MTB and AI in gastric cancer stage Ⅱ

**Supplementary table 3.** The discrepancies between the MTB and AI in gastric cancer stage Ⅲ.

**Supplementary table 4.** The discrepancies between the MTB and AI in gastric cancer stage Ⅳ.

**Supplementary table 1.** The discrepancies between the MTB and AI in gastric cancer stage Ⅰ

| Number | MTB | AI | |
| --- | --- | --- | --- |
|  |  | Recommended | Consideration |
| 1 | Surveillance | 5-FU / Leucovorin  Capecitabine S-1 |  |
| 2 | Surveillance | FOLFOX CapeOX Capecitabine+Radiation | 5-FU/Leucovorin + Cisplatin Capecitabine + Cisplatin 5-FU / Leucovorin Capecitabine S-1 5-FU/Leucovorin + Radiation |
| 3 | Surveillance | 5-FU / Leucovorin |  |
| 4 | Surveillance | FOLFOX CapeOX | 5-FU / Leucovorin + Cisplatin Capecitabine + Cisplatin 5-FU / Leucovorin Capecitabine  S-1 |
| 5 | Surveillance | FOLFOX CapeOX | 5-FU / Leucovorin + Cisplatin Capecitabine + Cisplatin 5-FU / Leucovorin Capecitabine S-1 |

Abbreviation; FOLFOX: fluorouracil/leucovorin/oxaliplatin; CapeOX: capecitabine/oxaliplatin

**Supplementary table 2.** The discrepancies between the MTB and AI in gastric cancer stage Ⅱ

| Number | MTB | AI | |
| --- | --- | --- | --- |
|  |  | Recommended | Consideration |
| 1 | Surveillance | 5-FU / Leucovorin Capecitabine S-1 |  |
| 2 | Surveillance | 5-FU / Leucovorin Capecitabine S-1 Capecitabine+Radiation | 5-FU / Leucovorin + Radiation |
| 3 | Surveillance | 5-FU / Leucovorin + Cisplatin  Capecitabine + Cisplatin  5-FU / Leucovorin; Capecitabine; S-1 |  |
| 4 | Surveillance | 5-FU / Leucovorin; Capecitabine | S-1 |
| 5 | S-1 | FOLFOX |  |

Abbreviation; FOLFOX: fluorouracil/leucovorin/oxaliplatin

**Supplementary table 3.** The discrepancies between the MTB and AI in gastric cancer stage Ⅲ.

| Number | MTB | AI | |
| --- | --- | --- | --- |
|  |  | WFO recommend | WFO consideration |
| 1 | S-1 | 5-FU/Leucovorin |  |
| 2 | XELOX | 5-FU/Leucovorin + cisplatin capecitabine + cisplatin 5-FU/Leucovorin Capecitabine  S-1 |  |
| 3 | S-1 | 5-FU/Leuocovorin + Radiation FOLFOX | 5-FU / Leucovorin  CapeOX Capecitabine + Radiation therapy |
| 4 | XELOX | Capecitabine + Radiation  5-FU / Leucovorin Capecitabine S-1; | 5-FU/Leucovorin + Radiation |
| 5 | S-1 | FOLFOX |  |
| 6 | S-1 | 5-FU/Leucovorin + Radiation FOLFOX | 5-FU / Leucovorin |

Abbreviation; XELOX: capecitabine/oxaliplatin; FOLFOX: fluorouracil/leucovorin/oxaliplatin; CapeOX: capecitabine/oxaliplatin

**Supplementary table 4.** The discrepancies between the MTB and AI in gastric cancer stage Ⅳ.

| Number | MTB | AI | |
| --- | --- | --- | --- |
|  |  | Recommended | Consideration |
| 1 | S-1 + cisplatin | FOLFOX | 5-FU / Leucovorin + Cisplatin Capecitabine + Cisplatin CapeOX |
| 2 | S-1 + cisplatin | FOLFOX | 5-FU / Leucovorin + Cisplatin Capecitabine + Cisplatin CapeOX FOLFIRI |
| 3 | S-1 + cisplatin | FOLFOX | FOLFIRI |
| 4 | S-1 + cisplatin | Dose Modified DCF;  FOLFOX | DOF; 5-FU / Leucovorin + Cisplatin  Capecitabine + Cisplatin CapeOX  FOLFIRI |
| 5 | S-1 + cisplatin | Dose Modified DCF;  FOLFOX | DOF 5-FU / Leucovorin + Cisplatin  Capecitabine + Cisplatin  CapeOX  FOLFIRI |
| 6 | S-1 + cisplatin | FOLFOX | 5-FU / Leucovorin + Cisplatin Capecitabine + Cisplatin CapeOX FOLFIRI |
| 7 | S-1 + cisplatin | FOLFOX | 5-FU / Leucovorin + Cisplatin Capecitabine + Cisplatin CapeOX FOLFIRI |
| 8 | S-1 + cisplatin | Dose Modified DCF FOLFOX | DOF 5-FU / Leucovorin + Cisplatin  Capecitabine + Cisplatin  CapeOX  FOLFIRI |
| 9 | S-1 + cisplatin | FOLFOX | 5-FU / Leucovorin + Cisplatin Capecitabine + Cisplatin CapeOX FOLFIRI |
| 10 | S-1 + cisplatin | Dose Modified DCF FOLFOX | DOF 5-FU / Leucovorin + Cisplatin  Capecitabine + Cisplatin  CapeOX  FOLFIRI |
| 11 | S-1 + cisplatin | FOLFOX | FOLFIRI |
| 12 | S-1 + cisplatin | FOLFOX | FOLFIRI |
| 13 | S-1 + cisplatin | FOLFOX | FOLFIRI |
| 14 | S-1 + cisplatin | FOLFOX | FOLFIRI |
| 15 | S-1 + cisplatin | FOLFOX | FOLFIRI |
| 16 | S-1 + cisplatin | FOLFOX | 5-FU / Leucovorin + Cisplatin Capecitabine + Cisplatin CapeOX FOLFIRI |
| 17 | S-1 + cisplatin | Dose Modified DCF FOLFOX | DOF 5-FU / Leucovorin + Cisplatin  Capecitabine + Cisplatin  CapeOX  FOLFIRI |
| 18 | S-1 + cisplatin | FOLFOX | 5-FU / Leucovorin + Cisplatin Capecitabine + Cisplatin CapeOX FOLFIRI |
| 19 | S-1 + cisplatin | Dose Modified DCF FOLFOX | DOF 5-FU / Leucovorin + Cisplatin  Capecitabine + Cisplatin CapeOX  FOLFIRI |
| 20 | S-1 + cisplatin | FOLFOX | FOLFIRI |
| 21 | S-1 + cisplatin | FOLFOX | FOLFIRI |
| 22 | S-1 + cisplatin | FOLFOX | DOF  CapeOX  FOLFIRI |
| 23 | S-1 + cisplatin | Dose Modified DCF FOLFOX | DOF 5-FU / Leucovorin + Cisplatin  Capecitabine + Cisplatin CapeOX  FOLFIRI |
| 24 | S-1 + cisplatin | FOLFOX | CapeOX  FOLFIRI |
| 25 | S-1 + cisplatin | Dose Modified DCF FOLFOX | DOF 5-FU / Leucovorin + Cisplatin  Capecitabine + Cisplatin CapeOX |
| 26 | S-1 + cisplatin | Dose Modified DCF FOLFOX | DOF 5-FU / Leucovorin + Cisplatin  Capecitabine + Cisplatin CapeOX |

Abbreviation; FOLFOX: fluorouracil/leucovorin/oxaliplatin; CapeOX: capecitabine/oxaliplatin; FOLFIRI: fluorouracil/leucovorin/irinotecan; DCF: docetaxel/cisplatin/fluorouracil/leucovorin; DOF: docetaxel/oxaliplatin/fluorouracil
